# Mining the Breast Cancer Proteome for Predictors of Drug Sensitivity

**DOI:** 10.4172/jpb.1000370

**Published:** 2015

**Authors:** Leslie C Timpe, Dian Li, Ten-Yang Yen, Judi Wong, Roger Yen, Bruce A Macher, Alexandra Piryatinska

**Affiliations:** 1 Department of Mathematics, San Francisco State University, San Francisco, California 94132, USA; 2 Department of Chemistry and Biochemistry, San Francisco State University, San Francisco, California 94132, USA

**Keywords:** Breast cancer cell lines, Chemotherapy, Targeted agents, Mass spectrometry, Statistical modeling, Lasso regression, Elastic net regression

## Abstract

Approximately 20 drugs have been approved by the FDA for breast cancer treatment, yet predictive biomarkers are known for only a few of these. The identification of additional biomarkers would be useful both for drugs currently approved for breast cancer treatment and for new drug development. Using glycoprotein expression data collected via mass spectrometry, in conjunction with statistical models constructed by elastic net or lasso regression, we modeled quantitatively the responses of breast cancer cell lines to ~90 drugs. Lasso and elastic net regression identified HER2 as a predictor protein for lapatinib, afatinib, gefitinib and erlotinib, which target HER2 or the EGF receptor, thus providing an internal control for the approach. Two additional protein datasets and two RNA datasets were also tested as sources of predictor proteins for modeling drug sensitivity. Protein expression measured by mass spectrometry gave models with higher coefficients of determination than did reverse phase protein array (RPPA) predictor data. Further, cross validation of the elastic net models shows that, for many drugs, the prediction error is lower when the predictor data is from proteins, rather than mRNA expression measured on microarrays. Drugs that could be modeled effectively include PI3K inhibitors, Akt inhibitors, paclitaxel and docetaxel, rapamycin, everolimus and temsirolimus, gemcitabine and vinorelbine. Strikingly, this modeling approach with protein predictors often succeeds for drugs that are targeted agents, even when the nominal target is not in the dataset.

## Introduction

The U.S. Food and Drug Administration has approved roughly two dozen drugs for breast cancer treatment, but only a few predictive biomarkers are currently available to guide their use. For example, levels of the estrogen or progesterone receptor have been found to predict sensitivity to compounds that interfere with estrogen receptor signaling, and, the over-expression of human epidermal growth factor receptor 2 (*HER2*) predicts sensitivity to pertuzumab, trastuzumab and lapatinib. Additional biomarkers would be of significant value for determining the sensitivity of tumors to drugs already approved for clinical use in breast cancer, as well as for new drug development [1,2].

What sort of data could yield accurate predictions of a patient’s response to a drug? Recent efforts in this area have focused on solving the simplified problem of predicting the responses of cancer cell lines, rather than of tumors [3–8]. This line of inquiry has explored a variety of predictor variables: gene mutations, copy number variation, methylation patterns, gene expression data [3–5,7,8], reverse phase protein array data [4,9], and receptor signaling networks [10]. In addition, researchers have begun to apply statistical and machine learning methods to evaluate and improve the identification of predictors [11–14]. Geeleher et al. [15] propose that combining cell line data with measurements of gene expression in patient tumor samples can be used to predict a patient’s response to a drug.

RNA isolated from tumors has attracted considerable interest as a source of predictors of drug response in several types of cancer [16,17]. To date these attempts have not achieved sufficient success for application in the clinic. Immunohistochemistry is now used to characterize the expression of the estrogen receptor, progesterone receptor and HER2 proteins in breast cancer, suggesting that protein expression data may be more useful than mRNA in predicting drug response.

Our research group has assembled a database of glycoproteins from 26 breast cancer cell lines [18,19]; for 22 of these cell lines drug response data is publicly available. We used liquid chromatorgraphy/tandem mass spectrometry (LC/MS/MS) to identify the proteins, and spectral counting to determine their relative expression levels. This dataset provides an opportunity to evaluate the usefulness of protein expression measured by mass spectrometry in modeling the sensitivities of the cell lines to drugs, and to compare the performance of various types of data in prediction.

The glycoproteins in our dataset are primarily secreted or plasma membrane proteins. The dataset is enriched for proteins that mediate contacts between epithelial cells, as well as for components of the basement membrane and extracellular matrix. We have shown that many of these proteins are expressed at different levels in malignant compared to non-malignant cell lines, and that the malignant cell lines are typically characterized by significantly lower levels of glycoprotein expression [18].

Large data sets describing the effects of various drugs on the growth of cancer cell lines have recently been generated for the purpose of accelerating the preclinical evaluation of new compounds [3–8]. One of these datasets describes the effects of 90 drugs on 70 breast cancer cell lines, and is the largest of the datasets with respect to the number of drugs and breast cancer cell lines [4]. Those authors measured the concentration of each drug that causes a 50% reduction in the proliferation of cells in culture (GI_50_). One striking finding is that the cell lines vary greatly in sensitivity to various drugs, in some cases by more than four orders of magnitude. While acquired resistance to chemotherapeutics or targeted agents is well recognized and is the subject of intensive study [20], the variation in sensitivities to these 90 drugs displayed by the cell lines in culture is not likely to be due to resistance acquired from previous exposure to these drugs; the patients from whose tumors the cell lines were derived would not have been treated with most of these drugs. Thus, there appears to be much intrinsic variability in the responses of these tumor-derived cell lines to drugs. If replicated in breast tumors, these intrinsic differences in sensitivity could explain some of the variability of patients’ responses to chemotherapeutic drugs or targeted agents.

We applied regularized regression to model the intrinsic sensitivities of cell lines to 90 drugs [4] using the glycoprotein dataset. For purposes of comparison, we also modeled the drug sensitivities using mRNA expression for mRNA species corresponding to the glycoproteins in our dataset using two mRNA datasets. Those datasets, both publicly available, are a microarray dataset [7] and an RNAseq dataset [4]. In addition we modeled the drug sensitivities using two publicly available protein datasets, ones not primarily based on glycoproteins [4,21].

## Materials and Methods

Our glycoprotein dataset includes 185 glycoproteins obtained from 22 breast cancer cell lines. Glycoproteins were collected using a protocol in which the first step, oxidation of the glycans using periodate, takes place on intact cells [19]. After cell lysis and enrichment for glycoproteins, the samples were subjected to LC/MS/MS to identify the proteins. Our protocol for glycoprotein enrichment and analysis by LC/MS/MS is described in detail elsewhere [19]. Relative quantitation was achieved by counting identified spectra (spectral counts) (Supplementary Information Table 1). The glycoprotein data is similar to that described previously [18,19], with seven additional cell lines (Supplementary Information Table 2). Collectively the data includes cell lines classified as luminal, basal, claudin-low, ER positive and HER2 overexpressing. With respect to these variables, the set of cell lines reflects much of the variety present in breast tumors.

Protein analyses were carried out on a Thermo LTQ ion trap mass spectrometer and a Thermo Q Exactive Orbitrap mass spectrometer. Spectral counts were used to determine relative expression levels of a glycoprotein in the various cell lines. Aliquots from the same glycoprotein sample produced similar results when analyzed on the two mass spectrometers, although less protein was required for the analyses carried out on the Orbitrap instrument. To combine samples from the two datasets, the data were plotted in a quantile-quantile plot, and a line was fit. Using the slope and intercept, the inverse transform was applied to the Q Exactive data, forcing it to have the same center and dispersion as the LTQ data. For four of the cell lines (HCC1395, HCC1428, HCC38 and MDAMB468) there were only Q Exactive data. Spectral counts for these cell lines were normalized to the LTQ data using household proteins. The seven proteins in the LTQ dataset with the lowest coefficient of variation were P20645, Q9BT09, P62937, Q16563, Q9BVK6 Q08722 and P07602. The Euclidean length of the spectral counts for these seven glycoproteins was calculated for both the LTQ data and the Q Exactive data, and the ratio was used to normalize all Q Exactive spectral count data. After combining LTQ and Q Exactive data, glycoproteins with fewer than 100 spectral counts over all the cell lines were dropped from the dataset. For purposes of regression analysis one spectral count was added to all values, and the base ten logarithm taken.

### RNA data

Exon array analysis is described in reference 7. The data are available from ArrayExpress (E-MTAB-181). These data include 19 of the cell lines in the glycoprotein dataset, and measurements of mRNA expression for 160 of the glycoproteins. The RNA seq dataset (Gene Expression Omnibus, GSE48216) is from reference 4. The subset analyzed here covers 19 cell lines and 184 of the proteins in the glycoprotein data.

### RPPA data

The data (47 cell lines) was used as provided in Additional Files Table 2 of reference 4.

### MRM data

The multiple reaction monitoring (MRM) mass spectrometry data (27 cell lines) are based on Table S4 of reference 21. The measurements were generated from results obtained by three laboratories, with three replicates taken at each site. Two peptides were measured per protein. In some cases the measured values fell outside the limits of quantitation, which are provided in the study. For use in the present project the replicates and the data from different sites were averaged. For each protein the peptide with the highest signal was selected. In cases for which numerical values were not provided, the appropriate upper or lower limit of quantitation was used. The final dataset describes 317 proteins (Supplementary Information Table 3). Base ten logarithms were used for regression.

### Penalized Regression

The methods used are elastic net regression and lasso regression [22]. Calculations were performed using the *glmnet* package in the R statistical programming language. One adjustable parameter, *λ*, sets the amplitude of the penalty term; a second parameter, *α*, is a weight that determines the mixture of *L1* and *L2* norm components in the penalty. Letting *α = 1* gives lasso regression, and *0 < α < 1* gives elastic net regression. For elastic net regression we incremented *α* from 0 to 1 in steps of 0.1. For each value of *α,* we found the best value of *λ* by cross validation (*cv.glmnet* function), using the mean squared error (MSE) to evaluate the fit of the model to the data. Plots of MSE as a function of *α* showed some instability from run to run, so we used the average of 10 runs. The value of *α* giving the lowest MSE was selected for the elastic net model. These values differed from drug to drug. We performed cross validation by leaving out all pairwise combinations of cell lines; for the glycoprotein dataset (22 cell lines) this is similar to 10-fold cross validation. We found the correlations between each of the 21 cross validation estimates of drug sensitivities for all cell lines and the observed sensitivity values, and finally averaged these correlations. Optimal values of *α* and *λ* were determined for each training set in the cross validation as described above.

## Results and Discussion

**Figure 1:**
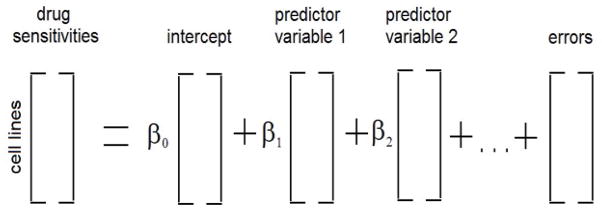
The regression model. One or more predictor variables are from the glycoprotein or other dataset.

Quantitative protein expression data may be more useful than mRNA data for predicting the responses of breast cancer cell lines to drugs. In this study we evaluated the ability of a glycoprotein dataset obtained via mass spectrometry to provide explanatory or predictor variables to fit measured drug sensitivities (Figure 1). The drug response profiles and the protein data are both quantitative, hence predicting the sensitivities of cell lines to various drugs implies modeling quantitative drug response data as a function of some number of quantitative predictor variables, i.e., it is a regression problem. There are 22 cell lines for which both drug sensitivity and spectral count data is available, and which are therefore suitable for regression modeling. There are 185 proteins in the glycoprotein dataset. With more predictor proteins than cell lines there is no unique solution to the regression problem for a given drug. However, there are methods, elastic net and lasso regression, to construct regression models and reduce the number of predictor variables to the more important ones in parallel [22]. Elastic net and lasso regression have been used previously for constructing regression models of the drug responses of cell lines using gene expression as predictor variables [3,5,11], and the performance of elastic net and ridge regression have been studied by simulation [12,14]. Here we used elastic net and lasso regression for each drug to develop models that fit cell line sensitivity to that drug.

Both elastic net and lasso regression reduce the number of predictor variables, but they do so to different extents. Elastic net regression models usually have more predictors than do the lasso models for the same drug, as a result the fits to the data are better. The disadvantage of the elastic net method is that with more variables the model may contain some predictors with little statistical or biological significance.

Rapamycin illustrates the differences between the two methods. The breast cancer cell lines in our sample vary in their sensitivity to rapamycin by more than four orders of magnitude. The model constructed using elastic net regression had 92 predictor variables, giving a very tight fit to the observed data. Models constructed using lasso regression showed some variability of results over 1000 separate runs, but three predictor proteins appeared in all models (Supplementary Information Table 4). The three predictors are HER2 (*ERBB2*, UniProt Accession number P04626), Disintegrin and metalloproteinase domain-containing protein 10 (*ADAM10,* O14672) and Junctional adhesion molecule A (*F11R*, Q9Y624). These three proteins are also among the 92 predictors identified in the elastic net model. Using the three proteins for ordinary least squares multiple regression gives a model in which the fitted drug sensitivities match the observed ones with a correlation coefficient of 0.91. It should also be noted that lasso regression identified HER2 as a predictor for sensitivity to everolimus and temsirolimus, two derivatives of rapamycin. While the lasso models generally do not fit the data as well as the elastic net models, they select fewer variables. For many drugs in this dataset the sensitivities could be fit well with 1–3 predictor variables (see below).

### Inhibitors of HER2 or the EGF receptor

Five of the drugs we examined were developed with the goal of inhibiting the epidermal growth factor receptor (EGFR), or its constitutively active variant, HER2. HER2 is present in the dataset and is over-expressed in three of the 22 cell lines. Lasso regression identifies HER2 as a predictor for the two HER2 inhibitors, afatinib (BIBW2992) and lapatinib, and also for two of the EGFR inhibitors, AG1478 and gefitinib. This finding serves as a positive control for the application of lasso regression to the glycoprotein data.

**Figure 2:**
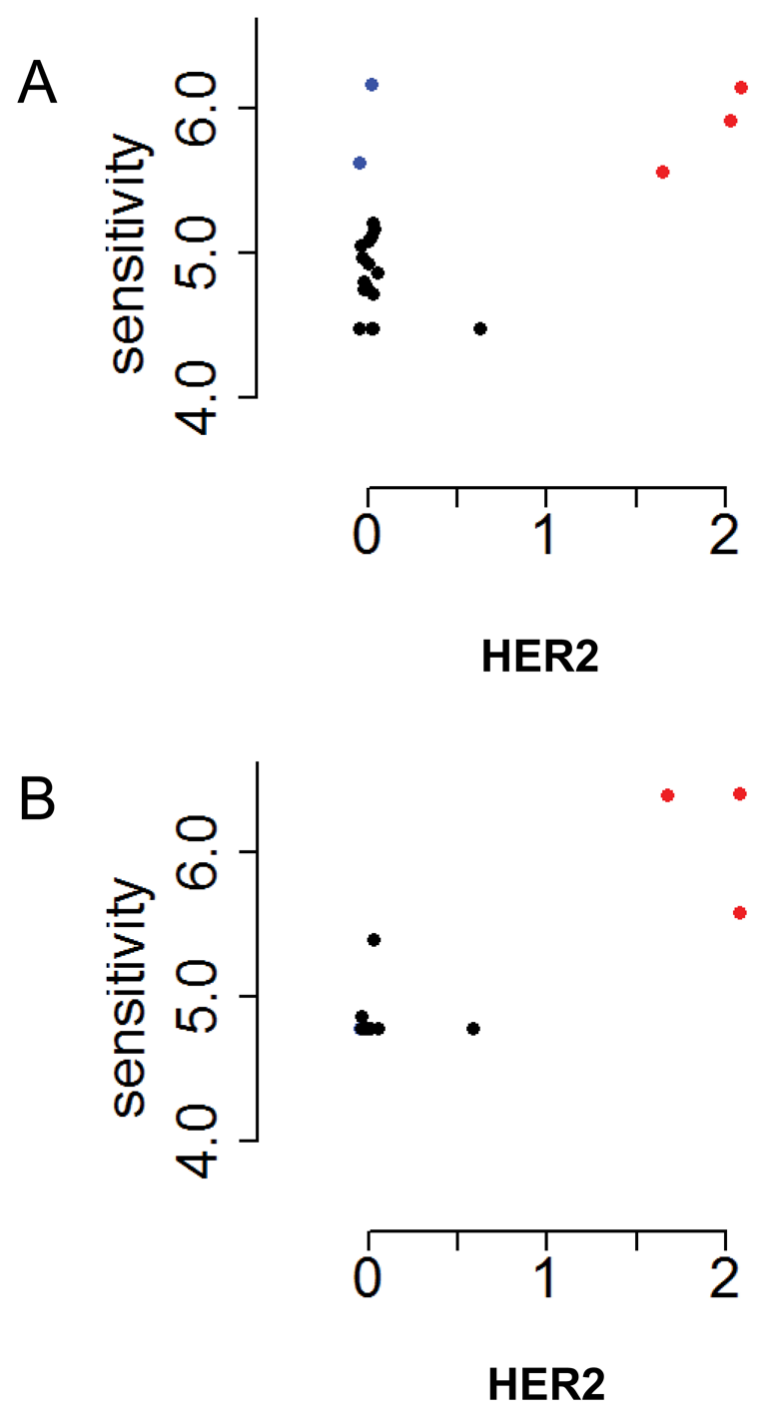
Drug sensitivity as a function of HER2 expression. Each point corresponds to a cell line. A. Gefitinib B. Lapatinib. Sensitivity (vertical axis) is the negative common logarithm of GI_50_, the drug concentration that inhibits proliferation by 50% (ref. 4). The horizontal axis is the common logarithm of the spectral counts, after adding 1 to each value. Red symbols: cell lines that overexpress HER2. Blue symbols: drug-sensitive cell lines that do not overexpress HER2.

The quantitative relationships between HER2 expression levels and drug sensitivities can be seen in the scatterplots in Figure 2. The HER2 over-expressing cell lines (red symbols) have comparatively high drug sensitivity (vertical axes) for gefitinib and lapatinib. Some cell lines with high sensitivity to gefitinib do not over-express HER2 (blue symbols). Scatterplots for AG1478, afatinib and erlotinib are similar to that of gefitinib, showing that over-expression of HER2 is associated with drug sensitivity, but in addition a few cell lines that do not overexpress HER2 are also drug sensitive.

### Regression models with multiple variables for EGFR/HER2 blockers

**Figure 3:**
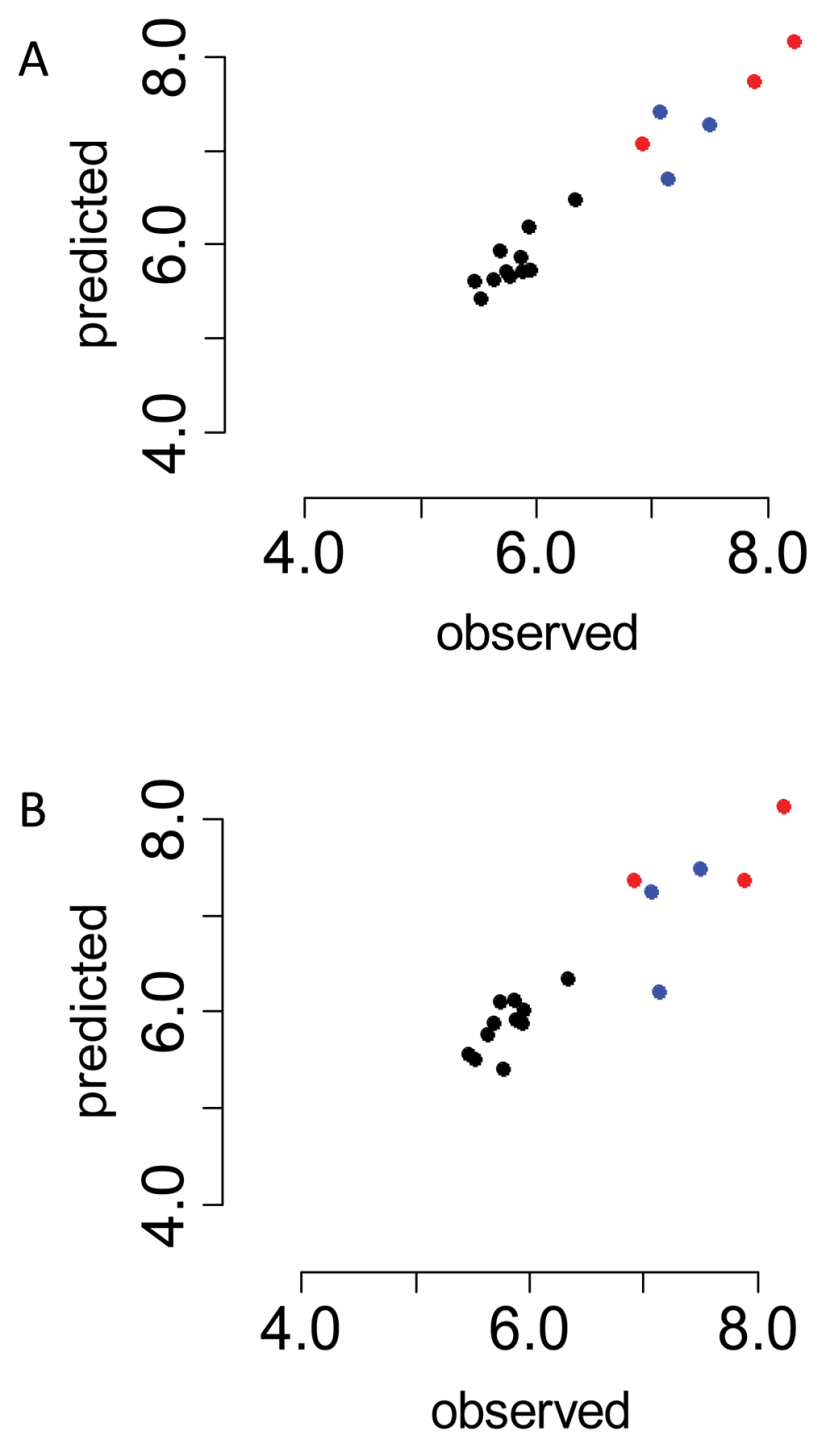
Comparison of predicted with observed sensitivities for afatinib (BIBW2992). Observed values of the drug sensitivities are plotted on the horizontal axes. A. Fitted values from elastic net modeling are plotted on the vertical axis. The predictors are HER2, SLC7A5, BST2, LAMB1, CTSB, CDH13, TCN1, SUSD2 and A2ML1. B. Lasso model. The four predictor variables from the lasso model are HER2, SLC7A5, BST2 and A2ML1. The fitted values (vertical axis) were constructed with these predictors using ordinary least squares regression. Red symbols: cell lines that overexpress HER2. Blue symbols: drug-sensitive cell lines that do not overexpress HER2.

By adding one or more predictor variables to HER2, it is possible to fit the sensitivities of the cell lines to gefitinib, AG1478, afatinib and erlotinib, where the relation between sensitivity and HER2 expression is not linear. The elastic net model for afatinib contained nine predictor variables, compared to four in the lasso model. For both models HER2 was one of the predictor variables. The elastic net model gives a somewhat tighter fit to the observed drug sensitivities (Figure 3). Both models place the afatinib-sensitive cell lines that do not over-express HER2 on the linear relation (blue symbols). In addition to HER2 (*ERBB2* or P04626), the lasso model included *SLC7A5* (large neutral amino acids transporter small subunit 1, Q01650), *BST2* (bone marrow stromal antigen 2, Q10589) and *A2ML1* (alpha 2 macroglobulin-like protein 1, A8K2U0); these are the four predictors identified most often in the lasso models. HER2 expression has a fairly high correlation with afatinib sensitivity, 0.65, but the SLC7A5, BST2 and A2ML1 have lower correlations, 0.61, −0.59 and 0.44, respectively. Part of their contribution is to enable the modeling of the afatinib-sensitive cell lines with normal HER2 expression (blue symbols) correctly. A model using only HER2 as a predictor would mistakenly suggest that the cell lines represented with blue symbols are not sensitive to afatinib.

### Models with one or three predictors

**Figure 4:**
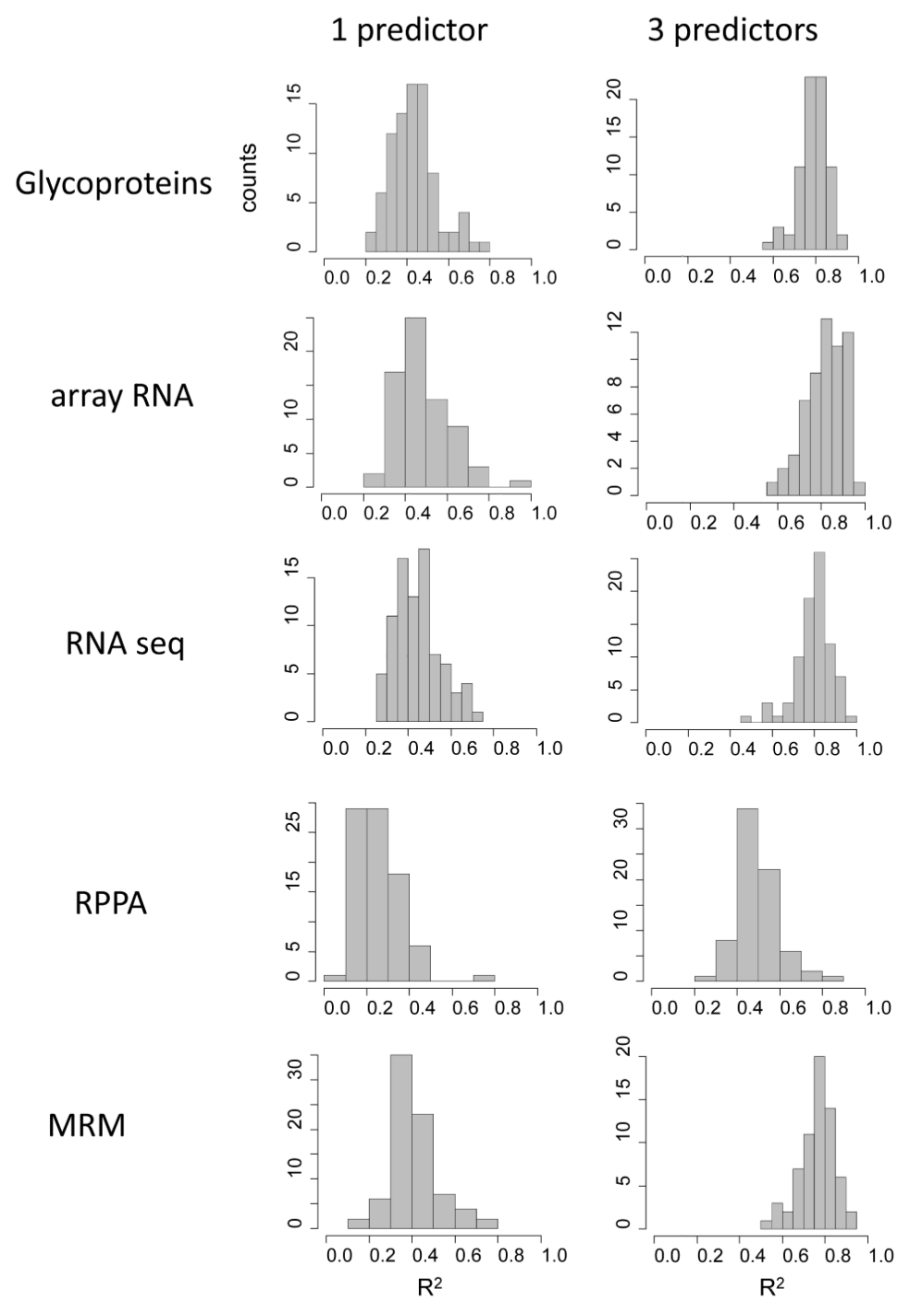
Frequency distributions of coefficients of determination (R^2^) for all single predictor models and all three-predictor models. For each drug the pool of candidate predictors was identified by lasso regression (Supplementary Information Table 4). The best (lowest MSE) one and three predictor models were identified using the Leaps and Bounds algorithm [23]. The coefficients of determination were found using ordinary least squares regression.

Lasso regression returned a model for 87 of 90 drugs (Supplementary Information Table 4). For each drug we identified the best model with one predictor protein, i.e. the one with the smallest mean squared error. The coefficients of determination (R^2^ values comparing the observed and fitted drug sensitivities) varied from 0.2 to nearly 0.8 (Figure 4). The frequency distribution of the coefficients of determination for the glycoprotein data is unimodal and approximately symmetrical, as expected from statistical theory. The distribution is skewed slightly to the right due to a few drugs for which we found an especially good model. Table 1 lists the top dozen drugs with their predictors. Interestingly, SLC7A5 (large neutral amino acids transporter small subunit 1), rather than HER2, is the best predictor for erlotinib, gefitinib and AG1478.

For each drug we found the best (lowest MSE) model with three predictors using the Leaps and Bounds algorithm [23]. The coefficients of determination are generally higher for the models with three predictor variables than they are for the one-predictor models (Figure 4). The average coefficient of determination for single predictor models was 0.44, whereas for the best three-predictor models it was 0.79. Clearly, increasing the number of predictors can greatly improve the performance of models in fitting the observed drug sensitivities.

The magnitude of the improvement may be exaggerated somewhat, due to the possibility of over fitting as the number of predictor variables increases and the best models are selected. Over fitting can occur when the model includes variables that by chance reduce the MSE for the model in the sample under study. A model that overfits would probably perform poorly on cell line data that was not used to construct the model. Predictor variables that contribute to overfitting may have little or no biological relevance to the problem of modeling drug sensitivities.

While increasing the number of predictor proteins from one to three may allow some overfitting, the proteins selected by the lasso algorithm as predictors often make biological sense. For example, the model illustrated in Figure 3B for afatinib included SLC7A (Q01650) and BST2 (Q10589) as predictors. Both have been identified independently in the context of breast cancer. SLC7A is one of five proteins in the Mammastrat test for patients at high risk for recurrence after hormone therapy [24]. A meta-analysis of gene expression datasets suggested that BST2 in breast tumors is a predictor for tumor size, aggressiveness and host survival [25]. Thus, it is plausible that these proteins may have value in modeling drug sensitivities. Increasing the number of predictors beyond one has the potential to model the observations better because it makes use of more information relevant to the drug or disease.

### Prediction error

We used all available cell line data to create and test the models presented so far. A more demanding and realistic approach would involve building a model based on some training data, then using it to predict drug response to other cell lines not in the training set. Given the time and expense involved in obtaining mass spectrometric data, it is more efficient to use a cross validation approach than to expand the data set further. In cross validation the cell lines are divided into a training set and a test set. A regression model built from the training data is then used to predict the drug sensitivities of the test set cell lines. The coefficient of correlation between the predicted and the observed drug sensitivities gives an idea of how well the modeling performs.

We carried out cross validation using elastic net regression on our glycoprotein dataset and on two related mRNA datasets: a microarray gene expression dataset that includes 74 drugs [7], and an RNA seq dataset that includes 90 drugs [4]. To compare directly the performance of mRNA and protein data, we trimmed the original mRNA datasets to contain only the mRNA corresponding to the glycoproteins. We then conducted a cross validation analysis, as described above. The two best performing models—for the Sigma Akt1, 2 inhibitor and gefitinib—used glycoprotein predictors (Table 2 and Supplementary Information Table 5). Overall the glycoprotein data and RNA seq data had better ability to model the observed drug sensitivities (higher correlation between observed and fitted values) than did the array gene expression data.

### Other protein datasets

We tested two other publicly available protein datasets for their ability to predict drug sensitivities: a reverse phase protein array (RPPA) dataset, which depends on antibody binding for quantitation [4], and a dataset obtained using mass spectrometry, resulting from a project to develop multiple reaction monitoring (MRM) assays for proteins in breast cancer cell lines [21].

In the RPPA dataset, the 70 proteins measured were pre-selected by the investigators on the basis of known linkage of the proteins to cancer, including proteins known to be important in the control of signaling pathways, cell proliferation and DNA repair.

In the MRM dataset, targeted assays were devised for 317 proteins in 30 breast cancer cell lines, of which 27 lines overlap with the drug response dataset. The proteins were selected by the authors of that study for differential expression across the cell lines. They are found in many cellular compartments and contribute to a wide range of biological processes. Quantitation was achieved by comparing the test signal intensity to that of a reference peptide labeled with a heavy, stable isotope. Only two proteins, HER2 and cadherin E, are common to the glycoprotein, RPPA and MRM datasets.

Figure 4 shows the distributions of the coefficients of determination for one and three predictor models built from the RPPA and MRM dataset. The distributions for models built using MRM predictors were similar to those built using glycoprotein, array mRNA and RNA seq predictors. In contrast, the RPPA data consistently gave models with lower coefficients of determination, with either one or three predictors.

The sensitivities of the cell lines to EGFR or HER2 blockers (AG1478, afatinib, erlotinib, gefitinib and lapatinib) were modeled effectively by both RPPA and MRM data, and these drugs often performed well in cross-validation (Table 2).

### Overall comparison of the five datasets

**Figure 5:**
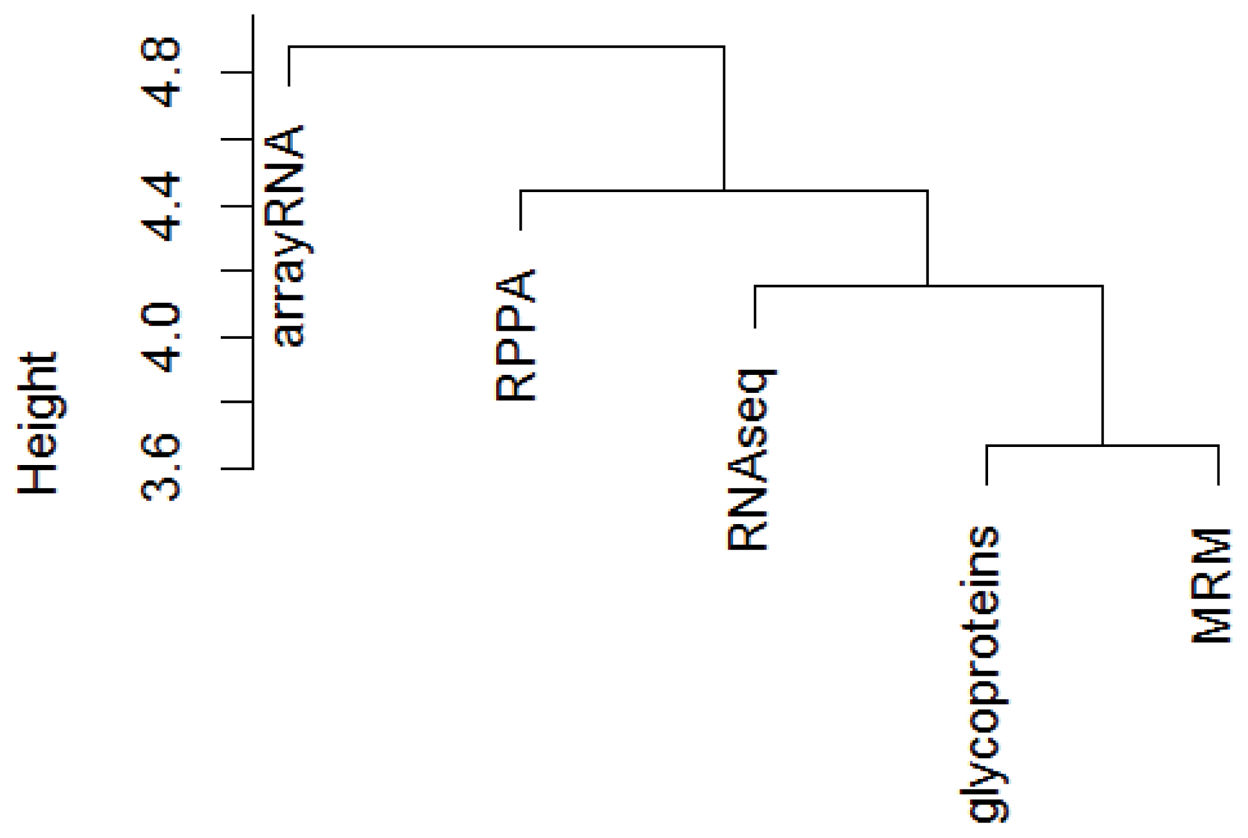
Dendrogram of datasets. The cross-validation data, some of which is displayed in Tables 2, were used to create and then cluster a distance matrix with the *dist()* and *hclust()* functions in R.

Summarizing the results so far, one dataset, the RPPA protein dataset, performed less well than the others in modeling using all cell lines, as judged from the distributions of the coefficients of determination (Figure 4). The array mRNA dataset performed less well than the others in cross validation (Table 2). Inspection of Table 2 shows that many of the same drugs among the top dozen, i.e. many drugs were modeled well using predictors from different datasets. How similar are the cross validation results of the five datasets to each other? The relationships are summarized in a dendrogram (Figure 5). The two mass spectrometry datasets (glycoprotein and MRM) [18,19,21] showed the strongest agreement with each other. The array RNA dataset is most distant from the others, with the RNA seq data giving results closer to those of the protein datasets. Notably, the drug sensitivity predictions of the glycoprotein and MRM datasets were closer to one another than were the predictions of the glycoprotein and RNA data. The glycoprotein and MRM datasets generally do not overlap in terms of proteins identified, whereas both RNA datasets contain only gene expression measurements of the glycoproteins.

### Examples of models: PI3K inhibitors

There are seven phosphatidylinositol-3-kinase (PI3K) inhibitors among the drugs. Three of them, BEZ235, GSK2126458 (omipalisib) and GSK 2119563 performed well in both the modeling (Supplementary Information Table 4) and the cross validation (Table 2). One protein, COL6A1, is a predictor for all three drugs. The two GSK inhibitors shared several inhibitors, including Suppressor of tumorigenicity 14 (ST14) and SPINT1, an inhibitor of ST14 and also of hepatocyte growth factor activator [26]. Finding common predictor proteins for different drugs in this class confirms our confidence in variable selection by lasso regression, and identifies proteins that may serve to predict the activity of PI3K inhibitors in patient samples.

### Rapamycin, everolimus and temsirolimus

Rapamycin, everolimus and temsirolimus are related compounds that block the mammalian target of rapamycin (mTOR); the cell lines varied in sensitivity to these drugs over 4.6, 3.3 and 3.7 orders of magnitude, respectively. mTOR is in the RPPA dataset, but was identified with very low probability as a predictor for these drugs (Supplementary Information Table 4). All three drugs can be modeled well with three glycoprotein predictors (Supplementary Information Figure 1). It can be seen that HER2 over-expressers are among the most sensitive cell lines. HER2 was the single common predictor for all three drugs. Everolimus is approved for use in patients with ER+, HER2- breast cancer, in combination with exemestane [27]. The cell line data suggests that HER2+ patients may also benefit from everolimus.

### Taxanes

The sensitivities of the cell lines to paclitaxel and docetaxel varied over smaller ranges than for rapamycin. For both drugs it was possible to find predictive models with high coefficient of determination (Supplementary Information Figure 2). Paclitaxel and docetaxel are similar chemically, hence it might be expected that they share some predictor proteins. Five common proteins were selected by most lasso models: FKBP4, USP5, MARS, CTSZ and ALDH7A1.

### Akt inhibitors

The drug list includes three AKT1 inhibitors: GSK2141795, Sigma AKT 1,2 inhibitor, and triciribine. The RPPA proteins include AKT (AKT1), AKTp473, and PDK1, a kinase that phosphorylates AKT. AKTp473 and PDK1 (or PDK1p241) were found in lasso regression models for all three drugs. A regression model with AKTp473 and PDK1 as predictors allowed the fitting of the GSK2141795 sensitivities with coefficient of determination (R^2^) = 0.52 (Supplementary Information Figure 3). By itself, PDK1 gives a better single predictor model (R^2^ =0.36) than does AKTp473 (R^2^ =0.20). For the Sigma inhibitor, PDK1 alone gives a model with R^2^ = 0.48; adding AKTp473 does not improve the model further. Modeling triciribine sensitivity failed with these two predictors. For the two drugs in which modeling succeeded, PDK1 is the more useful predictor even though AKT is the nominal target of the drugs.

### Gemcitabine and Vinorelbine

These drugs are used for patients who experience recurrence after treatment with the standard of care chemotherapy. As with rapamycin, the sensitivities of the cell lines to gemcitabine and vinorelbine spanned approximately four orders of magnitude. If this variation reflects the situation in patients’ tumors, there are patients who are highly sensitive to these drugs. The best model for gemcitabine (R^2^ = 0.77) appeared in the glycoprotein dataset, with predictors P17900 (Ganglioside GM2 activator), P28799 (Granulins) and P08842 (steryl sulfatase) (Supplementary Information Figure 2). The best model for vinorelbine (R^2^ = 0.85) was found in the MRM dataset, with predictors G6PD (Glucose-6-phosphate 1-dehydrogenase), HRSP12 (Ribonuclease UK114) and TPM4 (Tropomyosin alpha-4 chain).

### CDK inhibitors

Fascaplysin, NU6102, Olomoucine II and Purvalanol are inhibitors of cyclin-dependent kinases (CDKs) and are in the main drug database studied here. Palbociclib, another CDK inhibitor, has been analyzed elsewhere on breast cancer cell lines [28]. For all these drugs except Oloumucine II one or more cyclins were identified as predictors in a high proportion of lasso runs (RPPA data, Supplementary Information Table 4). The best model for palbociclib, with R^2^ = 0.79, was found in the MRM protein dataset using mitochondrial thioreduxin-dependent peroxidide reductase (PRDX3), acyl-amino acid releasing enzyme (APEH) and importin subunit alpha (KPNA2).

## Conclusions

Five datasets were compared in their abilities to provide predictors for regression modeling of drug sensitivities in breast cancer cell lines. We used two criteria for evaluating performance: the agreement between observed and predicted sensitivities (coefficient of determination), and prediction error estimated by cross validation. The glycoprotein and MRM datasets, obtained via mass spectrometry, and a RNA seq dataset performed best, with the glycoprotein and MRM datasets giving more consistent results in the cross validation.

Drugs that block the EGF receptor or HER2 were modeled well, at least partly because HER2 is in all the datasets. However, it is not necessary that a drug have its target in the data for this approach to work. For example, AKT and mTOR are not present in either the glycoprotein or MRM datasets, yet the Sigma Akt1,2 inhibitor and rapamycin were modeled accurately using predictors from these datasets. It appears that there is information useful for modeling drug sensitivity not just in the nominal targets of the drugs but also in the expression levels of other proteins.

These results show that it is possible to predict the responses of breast cancer cell lines to drugs, particularly when mass spectrometry has been used to quantify protein expression. Can the approach be extended to patient samples? One possibility would be to use targeted assays on tumor samples. The MRM dataset targets specific proteins; various methods for targeting proteins by mass spectrometry are currently under development [29]. Methods have also been developed for extracting proteins for mass spectrometry from formalin-fixed paraffin embedded (FFPE) samples [30], which are more readily available than fresh or frozen tissue, and which would be a convenient source of protein for targeted assays. Since patient outcomes for FFPE samples are known (e.g., whether the patient experienced a clinical response or an extended progression-free survival time), these outcomes would be modeled as response variables. Using protein expression levels, it may be possible to generate statistical models that predict patient response for many more drugs used in breast cancer.

## Supplementary Material

Supplementary file

## Figures and Tables

**Table 1:** The top twelve single predictor models for the glycoprotein dataset.

Drug	Accession Number	Gene Name	R^2^
Lapatinib	P04626	HER2	0.76
Sigma AKT1,2	P48960	CD97	0.70
Rapamycin	O14672	ADAM10	0.69
Gefitinib	Q01650	SLC7A5	0.67
GSK2141795	Q8IWA5	SLC44A2	0.66
Erlotinib	Q01650	SLC7A5	0.66
GSK2126458	P50897	PPT1	0.65
Ispinesib	P08195	SLC3A2	0.63
GSK1120212	P08648	ITGA5	0.60
Vorinostat	Q07954	LRP1	0.59
GSK1059615	P12830	CDH1	0.55
AG1478	Q01650	SLC7A5	0.53

**Table 2:** Top Performing Drugs in Cross Validation.

glycoproteins	R	RNA array	R	RNA seq	R	RPPA	R	MRM	R
AKT inhibitor	0.86	Cisplatin	0.58	Disulfiram	0.66	Lapatinib	0.79	Lapatinib	0.82
Gefitinib	0.79	AKT inhibitor	0.56	AKT inhibitor	0.62	Erlotinib	0.79	AKT inhibitor	0.77
GSK1059868	0.57	TCS 2312	0.55	OlomoucineII	0.59	BIBW2992	0.68	Rapamycin	0.62
GSK2126458	0.56	GSK2119563	0.46	Bosutinib	0.58	CPT 11	0.67	Docetaxel	0.58
Erlotinib	0.54	GSK2126458	0.44	GSK1059868	0.57	AKT inhibitor	0.67	AG1478	0.57
BEZ235	0.53	Erlotinib	0.42	GSK461364	0.54	AZD6244	0.58	BIBW2992	0.56
Rapamycin	0.53	CGC 11047	0.4	GSK2141795	0.53	Everolimus	0.56	GSK1070916	0.52
GSK2119563	0.44	Fascaplysin	0.39	GSK1120212	0.51	NU6102	0.54	PF 3814735	0.52
Vorinostat	0.41	GSK923295	0.36	Etoposide	0.48	LBH589	0.49	Sunitinib	0.48
Lapatinib	0.35	Etoposide	0.3	PF 4691502	0.48	Triciribine	0.47	PF 4691502	0.43
Ispinesib	0.29	LBH589	0.3	Gemcitabine	0.47	GSK461364	0.45	GSK2119563	0.43
AZD6244	0.29	AS 252424	0.25	AZD6244	0.39	GSK2126458	0.43	Tykerb:IGF1R	0.4
